# Effects of Nitrogen Addition and Fire on Plant Nitrogen Use in a Temperate Steppe

**DOI:** 10.1371/journal.pone.0090057

**Published:** 2014-03-03

**Authors:** Hai-Wei Wei, Xiao-Tao Lü, Fu-Mei Lü, Xing-Guo Han

**Affiliations:** 1 State Key Laboratory of Forest and Soil Ecology, Institute of Applied Ecology, Chinese Academy of Sciences, Shenyang, China; 2 Graduate University of Chinese Academy of Sciences, Beijing, China; 3 State Key Laboratory of Vegetation and Environmental Change, Institute of Botany, Chinese Academy of Sciences, Beijing, China; Beijing Forestry University, China

## Abstract

Plant nitrogen (N) use strategies have great implications for primary production and ecosystem nutrient cycling. Given the increasing atmospheric N deposition received by most of the terrestrial ecosystems, understanding the responses of plant N use would facilitate the projection of plant-mediated N cycling under global change scenarios. The effects of N deposition on plant N use would be affected by both natural and anthropogenic disturbances, such as prescribed fire in the grassland. We examined the effects of N addition (5.25 g N m^−2^ yr^−1^) and prescribed fire (annual burning) on plant N concentrations and N use characters at both species and community levels in a temperate steppe of northern China. We found that N addition and fire independently affected soil N availability and plant N use traits. Nitrogen addition increased aboveground net primary productivity (ANPP), inorganic N, and N uptake, decreased N response efficiency (NRE), but did not affect biomass-weighed N concentrations at community level. Prescribed fire did not change the community level N concentrations, but largely decreased N uptake efficiency and NRE. At the species level, the effects of N addition and fire on plant N use were species-specific. The divergent responses of plant N use at community and species levels to N addition and fire highlight the importance of the hierarchical responses of plant N use at diverse biological organization levels to the alteration of soil N availability. This study will improve our understanding of the responses of plant-mediated N cycling to global change factors and ecosystem management strategies in the semiarid grasslands.

## Introduction

Nitrogen (N) availability is the dominant limiting factor for primary productivity in most terrestrial ecosystems [1]–[4]. Consequently, the responses of plants to the variation in soil N availability would have great implications for plant growth and species competition. It is well established that plant N economy plays an important role in affecting plant productivity and ecosystem N cycling [5], [6]. Both plant N concentration and N response efficiency (NRE) are widely used to evaluate the responses of plant N economy to changes of soil N availability. Changes of plant N concentration have great implications for their N use efficiency (NUE), which is generally defined as the dry mass productivity per unit N taken up from the soil [7]. N response efficiency is the amount of biomass produced per unit of plant available N in the soil [8]–[10]. While plant N concentration is a indicator that integrates plant physiological and morphological responses to soil N availability, NRE is an index that integrates ecological and biogeochemical responses of plants. There is increasing empirical evidence that both plant N use indices should be simultaneously examined as they involve different processes linking the plant-soil system [11].

Both increasing atmospheric N deposition and global warming would enhance soil N availability in terrestrial ecosystems [12], [13], which exerts positive effects on plant growth and primary productivity [1], [14]. It should be noted that, however, the relationship between increased N availability and plant N use characters may not be straightforward in natural ecosystems. For instance, the changes of NUE in response to N availability would be the balance between the responses of its two components, NPP and the amount of N taken up by plants. Similarly, the responses of NRE would be determined by the relative changes of NPP and soil N availability. A decrease of NRE in response to N addition means that N addition shows relatively stronger impacts on soil N availability than NPP. Previous studies showed that N addition generally resulted in an increase of plant N concentration and a decrease of NRE at species level [8], [11], [15], [16]. It has been suggested that NRE would be a more representative measurement of plant responses to increasing soil N availability [11].

Most of previous studies mainly focused on changes of foliar N characters along nutrient availability gradients [6], which may not well mirror what is happening at higher biological organization levels. For example, there is significant variation of N concentrations between leaf and stem and also between aboveground and belowground parts at the individual plant level [17]–[18]. Furthermore, natural communities are always composed of plant species with various nutrient use strategies [19] and different nutrient limitation status [20]. There is increasing evidence that the response of plant N use to changes of nutrient availability would vary significantly among diverse biological scales, from foliar to community level [11].

Environmental disturbances and ecosystem management strategies may also affect plant N use through their impacts on soil N availability [21]–[23] and community composition [24]–[26]. Moreover, they have the potential to alter the magnitude and direction of the impacts of N addition on plant N use [27]. Fire, as an anthropogenic or natural disturbance, exerts great control on community composition and ecosystem functioning in several terrestrial ecosystems, including grassland [25], [28], [29]. Reduction in fire frequency is considered as one of the major reasons for the increases of woody plant abundance in grasslands all over the world [26], [30]. Prescribed burning is used as a strategy to prevent shrub encroachment in grasslands [31]. Results from a meta-analysis showed that short-term fire would enhance soil N availability while long-term repeated fire would lead to a decrease of soil N [21]. Similarly, the effects of fire on plant N status and use characters would also depend on its frequency. In the short-term, fire would increase plant NUE due to its stimulation on photosynthesis [32], whereas plant NUE would decrease under long-term repeated burning [22]. Given the interactive effects of fire and N addition on soil nutrient availability and plant production as observed in other studies [27], it is reasonable to expect that they may interact to affect plant N use. However, empirical evidence for their interactive effects on plant N use is still scarce.

The main objective of this study was to determine the effects of N addition, fire, and their interaction on plant N concentrations and N use characters at both species and community levels. We conducted a manipulative experiment in a temperate steppe in northern China, which is a typical vegetation type in the Eurasian continent. Fire was an important natural disturbance factor before the 1980s in northern China, with a frequency of once very 3–5 years [33]. In previous studies, we found that N addition and fire exert influence on plant nutrient resorption [34] and C∶N∶P stoichiometry [35], [36]. We tested the following hypotheses: (1) N addition would enhance plant N concentrations, but reduce plant N uptake efficiency and NRE. (2) Fire would also increase plant N concentrations, but decrease plant N uptake efficiency and NRE, as the short-term fire (annual burning for two years) usually enhance soil N availability. Furthermore, we expected that the responses of plant N use at species level would be different from that at community level.

## Materials and Methods

### Site description

This study was conducted near the Inner Mongolia Grassland Ecosystem Research Station (IMGERS, 116°42′E, 43°38′N), which is located in the middle reach of the Xilin River, northern China. Long-term (1970–2007) meteorological data indicated that the mean annual precipitation was approximately 345 mm and mean annual temperature was 0.3°C. The length of growing season is about 150 days. The soil of this site is classified as Haplic Calcisols according to the Food and Agriculture Organization classification. Mean bulk density (top 10 cm) is 1.3 g cm^−3^ and pH is 7.4. The vegetation is dominated by *Leymus chinensis* (Trin.) Tzvel., *Stipa grandis* P. Smirn., *Cleistogenes squarrosa* (Trin.) Keng., *Caragana microphylia* Lam., *Carex korshinskyi* Kom., and *Potentilla bifurca* L.

### Experimental design

This study was conducted as part of the GFE (Grassland Fire Experiment) experimental setup near IMGERS. All necessary permits were gained from Institute of Botany, Chinese Academy of Sciences and local government before the beginning of this study. The design consists of nine blocks distributed across a grassland field, with each block containing a set of 10 m×10 m plots representing fully crossed treatments of fire frequency, N addition, and mowing frequency. Plots were separated by 1 m buffers. The experiment setup was established in 2005, with all burning, N addition, and mowing treatments were started since 2006. Burning was carried out in early or late April each year before the start of growing season. Nitrogen in the form of NH_4_NO_3_ was added in the rainy days of late June each year. Only the treatments of two burning frequencies (never burned vs annual burning) and two N addition levels (0 vs 5.25 g N m^−2^ yr^−1^) that without mowing in 9 blocks were used in this study, with 4 treatments ×9 replications = 36 plots in all.

### Sample collection and chemical analysis

Aboveground net primary production (ANPP) was determined by clipping all plants in randomly located 1 m×1 m quadrats in each plot during the peak of growing season in August 2007. All plant samples were oven-dried at 70°C for 48 h, and then weighed. Samples of each species per plot were combined and ground to pass through a 40-mesh sieve using a mechanical mill (Retsch MM 400, Retsch GmbH & Co KG, Haan, Germany). Total N concentrations were determined colorimetrically by the Kjeldahl acid- digestion method with an Alpkem auto-analyzer (Kjektec System 1026 distilling unit, Sweden) after extraction with sulfuric acid.

Three soil cores (topsoil, 0–10 cm) were sampled in each of those quadrats following plant sampling with a metal tube (5 cm in diameter) and pooled to obtain one composite soil sample per quadrat. To measure soil water content, a soil subsample from each quadrat was weighed before and after being oven dried at 105°C for 48 h. Soil inorganic N availability was determined by extracting 10 g of fresh, root-free soil with 50 mL 2M KCl. The soil-extractant mixture was shaken for 1 h and then filtered (Whatman No. 1 filter paper). NH_4_^+^-N was measured with the salicylate method while NO_3_^−^-N was measured using the cadmium reduction method on a FIAstar 5000 Analyzer (Foss Tecator, Denmark). Total soil inorganic N was the sum of NH_4_^+^-N and NO_3_^−^N.

### Calculations and statistical analysis

The community level biomass-weighed N concentration was calculated as:
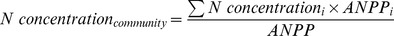
(1)Where *i* is the *i*th species of *n* species in the community.

N uptake efficiency was calculated as the proportion of available N in the soil that was taken up by the plants [8]

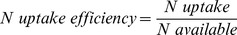
(2)NRE was calculated ANPP relative to the pool of available soil N [8]–[10]:
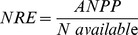
(3)The Kolmogorov-Smirnov test was used to test for data normality. Two-way ANOVAs with fire and N addition as the main factors were performed to examine main and interactive effects on soil inorganic N, soil water content, ANPP, N concentrations, N uptake, N uptake efficiency, and NRE at community level. Two-way ANOVAs were used to examine the effects of N addition and fire on the plant N use characters of five dominant species at species level. All statistical analyses were performed using SPSS software (version 13.0 for windows, SPSS Inc., Chicago, IL, USA).

## Results

### Community level responses

**Figure 1:**
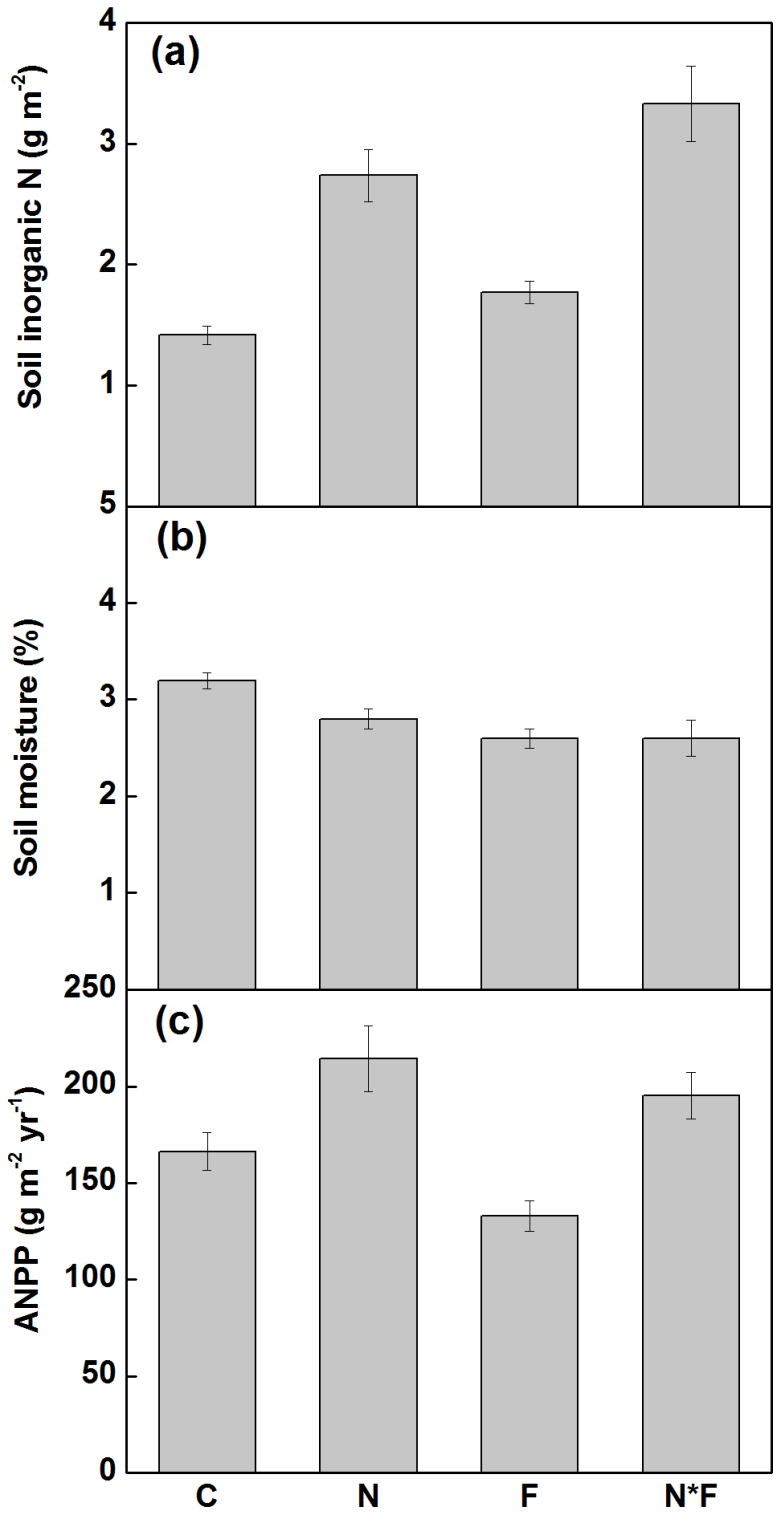
The effects of nitrogen addition and fire on (a) soil inorganic N concentrations, (b) soil water content, and (c) ANPP of plant community in a temperate steppe. C, control; N, nitrogen addition; F, fire; N*F, both N addition and fire. Data are adjusted means ± 1SE, where *n* = 9 for all treatments.

Nitrogen addition and fire significantly enhanced soil inorganic N by 94.2% (*P*<0.001) and 25.8% (*P*<0.05), respectively (Fig. 1a, Table 1). Fire significantly reduced soil water content (*P*<0.001), whereas N addition did not affect soil water content (Fig. 1b, Table 1). Nitrogen addition significantly increased ANPP by 36.3% (*P*<0.001; Fig. 1c, Table 1), whereas fire reduced ANPP by 14.3% (*P*<0.05; Fig. 1c; Table 1).

**Table 1:** Results (F-values) of two-way ANOVAs on the effects of nitrogen addition (N), fire (F), and their interaction on soil inorganic N, soil water content (SWC), ANPP, N concentration, N uptake, N uptake efficiency, and NRE at both community and species level in a temperate steppe.

Factor	Inorganic N	SWC	ANPP	N concentration	N uptake	N uptake efficiency	NRE
Community-level							
N	53.5***	2.5	24.2***	0	12.4**	7.9**	13.6***
F	6.0*	10.3**	6.2*	3.5	0.2	4.7*	20.7***
N*F	0.4	2.6	0.3	1.5	0.3	0.1	3
Species-level							
*Agropyron cristatum*							
N	-	-	3.6	0	4.6*	11.9**	10.9**
F	-	-	0.7	6.3*	0.1	1.5	5.8*
N*F	-	-	0.2	6.5*	0.5	0.2	2.5
*Carex korshinskyi*							
N	-	-	13.1**	3.5	13.5***	2.4	5.1*
F	-	-	16.3***	7.9**	8.4**	15.4***	27.6***
N*F	-	-	2.2	0.1	1.5	0.1	0.3
*Cleistogenes squarrosa*							
N	-	-	0.6	0.5	1	11.2**	12.5**
F	-	-	0.3	2.9	0	0.7	2.1
N*F	-	-	5.2*	2	5.4*	1.3	0.4
*Leymus chinensis*							
N	-	-	5.2*	2.3	0.9	7.0*	6.4*
F	-	-	0	6.7*	2.3	0.2	2.3
N*F	-	-	0.4	0	0	0	0
*Stipa grandis*							
N	-	-	0.1	1.9	0	5.1*	5.0*
F	-	-	3.8	18.2***	1.4	2.3	4.5
N*F	-	-	2	12.3**	3.7	0.7	0.1

The F-values are presented, together with their level of significance.

*P<0.05;

**P<0.01;

***P<0.001.

For all variables error *df* = 36.

**Figure 2:**
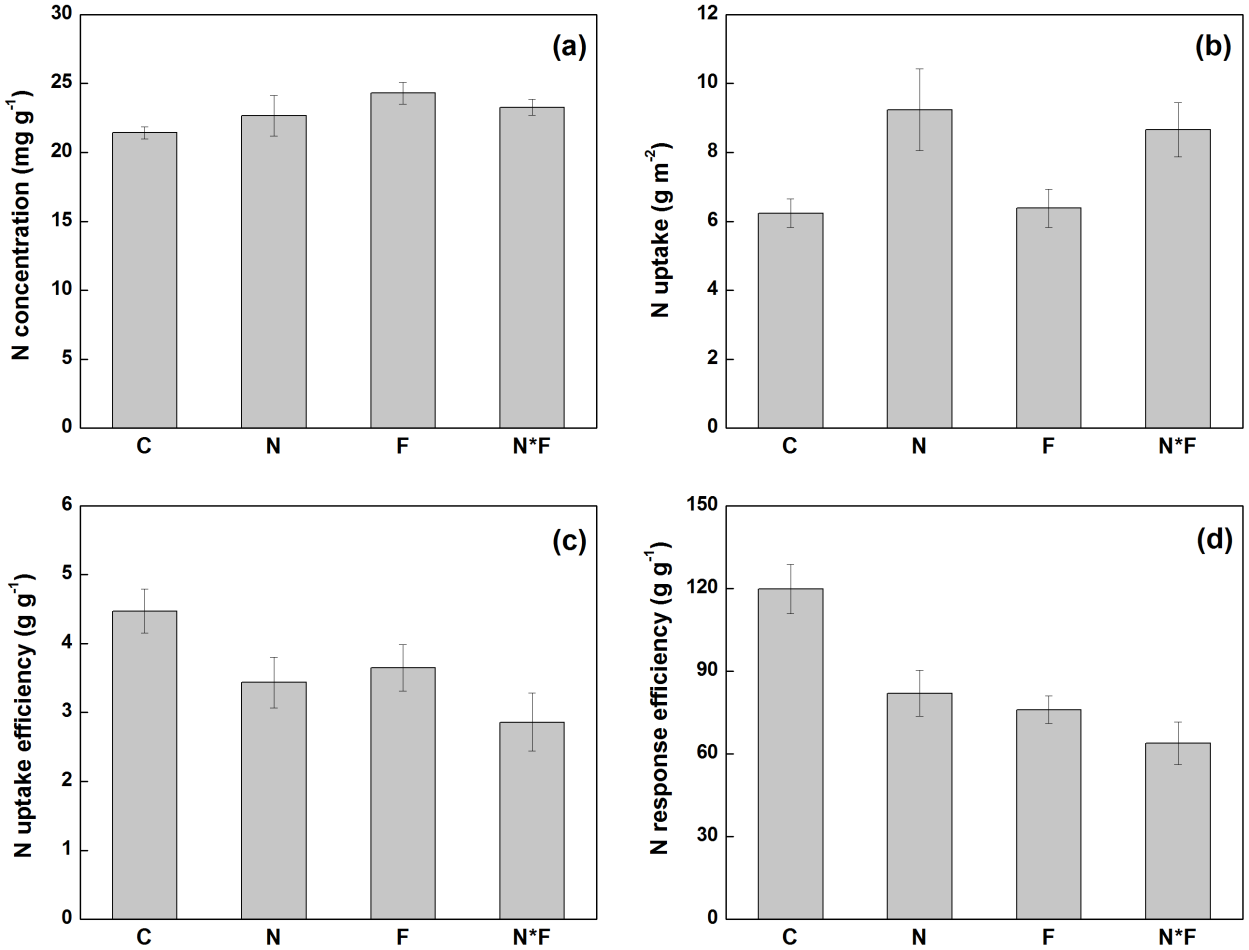
The effects of nitrogen addition and fire on (a) biomass-weighed N concentration, (b) N uptake, (c) N uptake efficiency, and (d) N response efficiency of plant community in a temperate steppe. C, control; N, nitrogen addition; F, fire; N*F, both N addition and fire. Data are adjusted means ± 1SE, where *n* = 9 for all treatments.

Both N addition and fire had no impacts on biomass-weighed N concentrations at community level (Fig. 2a; Table 1). N addition enhanced plant N uptake by 41.6% (*P*<0.01; Fig. 2b; Table 1), while fire had no effects on plant N uptake (Fig. 2b; Table 1). Both N addition and fire decreased N uptake efficiency by 22.9% (*P*<0.01) and 18.2% (*P*<0.05; Fig. 2c; Table 1). NRE was significantly reduced by both N addition (31.5%, *P*<0.001, Fig. 2c; Table 1) and fire (36.6%, *P*<0.001, Table 1). There was no interaction between N addition and fire for all variables (Table 1).

### Species level responses

**Figure 3:**
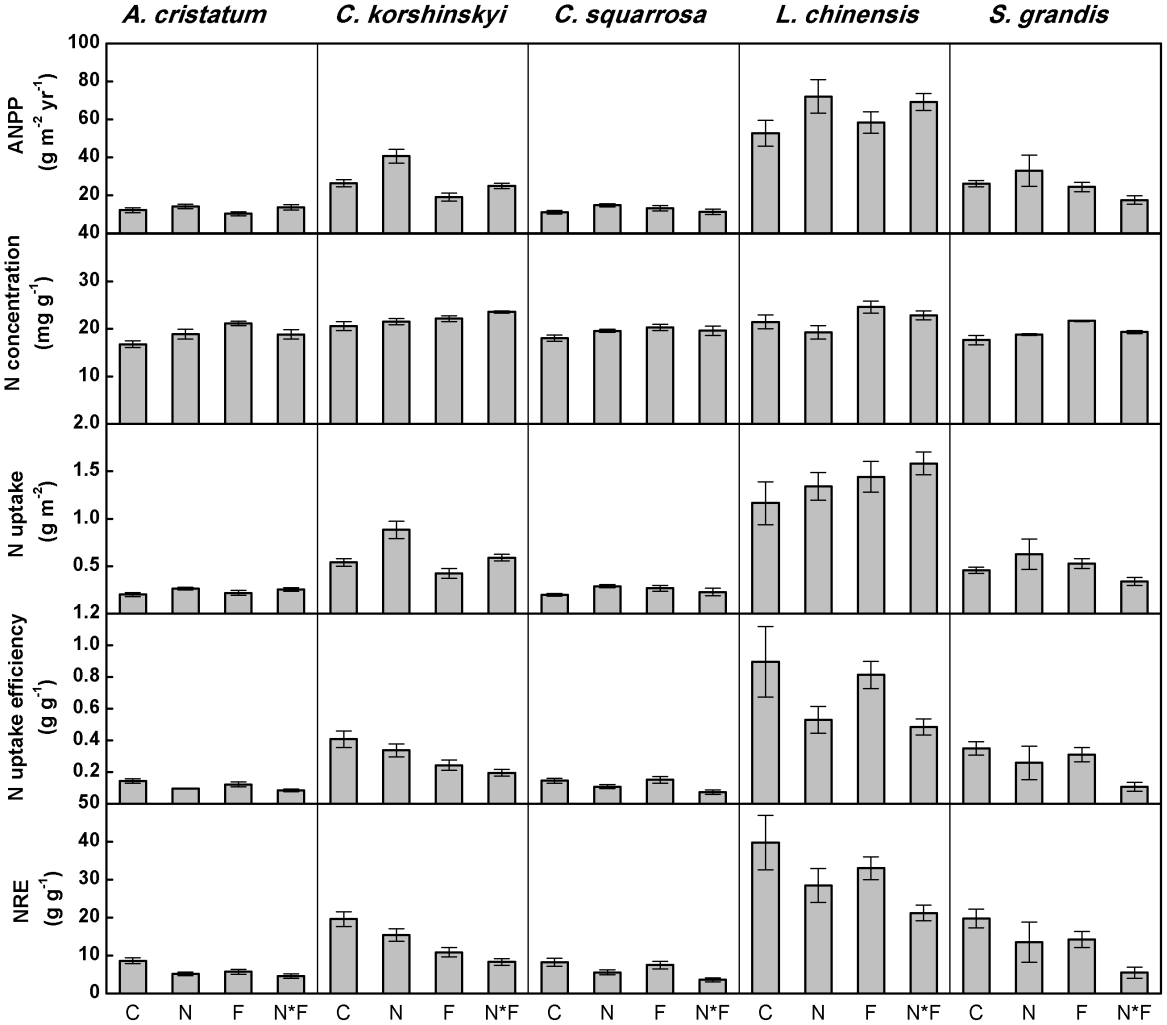
The effects of N addition and fire on plant N use of dominant species in a temperate stepp. C, control; N, nitrogen addition; F, fire; N*F, both N addition and fire. Data are adjusted means ±1SE, where *n* = 9 for all treatments.

Nitrogen addition significantly enhanced the ANPP of *C. korshinskyi* (*P*<0.01) and *L. chinensis* (*P*<0.05; Table 1; Fig. 3), but showed no impacts on that of *A. cristatum*, *C. squarrosa*, and *S. grandis*. When the species were analyzed individually, plant N use varied greatly among five dominant species (Fig. 3). N addition had no impacts on N concentration for all species, while fire significantly increased N concentration of all species except for *C. squarrosa* (Table 1; Fig. 3). N addition and fire interacted to affect N concentration of *A. cristatum* (*P*<0.05) and *S. grandis* (*P*<0.01; Table 1; Fig. 3). While N addition significantly enhanced N uptake in two out of all the five species (*A. cristatum* and *C. korshinskyi*), fire significantly reduced N uptake of *C. korshinskyi* but did not affect that of the other species (Table 1; Fig. 3).

Nitrogen addition significantly decreased N uptake efficiency of all species (*P*<0.05) except for *C. korshinskyi* (Table 1, Fig. 3), while fire only decreased the N uptake efficiency of *C. korshinskyi* (*P*<0.001). N addition significantly decreased NRE for all species (*P*<0.05), whereas fire only decreased that of *A. cristatum* (*P*<0.05) and *C. korshinskyi* (*P*<0.001). For all the species, there was no interactive effect between N addition and fire on NRE (Table 1).

## Discussion

Results from this study showed that N addition and fire directly affected soil N availability, ANPP, plant N uptake and use traits at both species and community levels. We found no interaction between N addition and fire in affecting plant N use. Generally, N addition increased ANPP and plant N uptake, decreased N uptake efficiency and NRE, with no significant effect on plant N concentrations. In contrast, fire showed positive effects on plant N concentrations (especially at species level), negative effects on NRE, and neutral effects on plant N uptake.

We found that both N addition and fire increased soil inorganic N concentrations. A meta-analysis showed that the soil inorganic N would increase immediately after fire and then gradually declined to the level before fire within one year [21]. All the burning plots had been burned for two years before soils were sampled in this study. Our results suggest that short-term fire would stimulate soil N turnover in the temperate steppe. While N is assumed to be the primary limiting nutrient in this ecosystem [14], we found negative effects of fire on ANPP even it stimulated soil N availability. We suspect that water availability would play an importantly in mediating the effects of fire on ANPP in this semiarid grassland. Fire significantly reduced soil water content in this study. In the same experimental setup, Zhou et al. [33] also found that the soil moisture in unburned plots was significantly higher than that in the burned plots during the growing season. Furthermore, it has been shown that water addition instead of N addition significantly increased ANPP, indicting water availability would be the dominant limiting factor for plant growth in this ecosystem [37]. Consequently, it is reasonable to speculate that reduction of soil water availability after burning would account for the negative effects of fire on community ANPP as observed in the present study.

Nitrogen addition would elevate soil inorganic N availability and thus increase ANPP, which had been generally tested and confirmed by previous studies [1], [14], [16]. Similarly, our results showed that N addition (5.25 g N m^−2^ yr^−1^) significantly enhanced soil inorganic N by 94.2%, and increased ANPP at both species and community levels. In the same area, Bai et al. [14] reported that N addition, even as low as 1.75 g N m^−2^ yr^−1^, would significantly increase ANPP. It is notable that, however, the effects of N addition on ANPP would be largely dependent on soil water availability [37]. The positive effects of N addition on the growth of dominant species and the ANPP of whole community as observed in this study indicated that primary production was limited by N availability in this area. Given the projected increasing atmospheric N deposition, the primary productivity would be facilitated following the increasing N availability. We found no interactive effect of N addition and fire on primary production at community level, implying that the positive effects of N deposition on primary production would not be affect by natural fire or prescribed burning in the temperate steppe of northern China.

Our results showed that N addition significantly increased plant N uptake by 41.6%, whereas fire had no effects on plant N uptake. The positive effects of N addition on plant N uptake were resulted from the enhancement of ANPP, as we found no impacts of N addition on plant N concentrations at both species and community levels. Previous studies showed that fire would increase N concentrations in plant tissues [27]. In this study, we found two-year annual fire significantly enhanced biomass-weighed plant N concentrations at community level, which is consistent with the results of our previous study which was carried out in the same ecosystem [35]. Due to the offset between the increases of plant N concentration and the decreases of ANPP following fire, annual burning showed no significant effects on plant N uptake. Fire showed negative influence on N uptake efficiency as it had no effects on plant N uptake but enhanced soil inorganic N. It has been reported that N addition would decrease N uptake efficiency, as the increases of N uptake cannot commensurate with the increases of soil inorganic N in a peatland and a managed grassland [11], [15]. Similarly, we found negative effects of N addition on plant N uptake efficiency at community level in this temperate steppe.

Previous studies reported that NUE often decreased with the increase of soil N availability [6], [9], [19], but not in other studies [38]–[40]. Contrary to our hypothesis, results from this study showed that N addition did not affect plant N concentrations at both species and community levels, implying that two-year N addition with the rate of 5.25 g N m^−2^ yr^−1^ did not affect NUE of plants in this temperate steppe. Our results were consistent with Reich et al. [41], who reported that increasing N availability did not change NUE in the forest ecosystem, but were different from Iversen et al. [11], who found that N addition (6 g N m^−2^ yr^−1^) resulted in a decrease of NUE in peatlands. In temperate grassland, Yuan et al. [40] found that the responses of NUE to N addition were dependent on N addition rates. On the one hand, in our study, the N addition rate was 5.25 g N m^−2^ yr^−1^, which might be not enough to induce a decrease of NUE. On the other hand, our results indicate that the efficiency of temperate steppe to use N would be consistent in the face of increasing N deposition, at least at the rate of 5.25 g N m^−2^ yr^−1^. Our results that two years annual burning significantly enhanced biomass-weighed N concentrations at community level, indicating that fire would have negative effects of NUE in this community. The enhancement of plant N concentrations would have great means for litter quality and decomposition, as the litter decomposition rate and nutrient release are usually positive related to the N concentration in the litter [5], [24], [42].

Consistent with our hypothesis and findings from other studies [11], [15], our results showed that N addition reduced NRE. Based on a natural soil N availability gradient in the semiarid grassland, Yuan et al. [43] found that NRE decreased monotonically with increasing N availability. The lower magnitude of the responses of ANPP to N addition than that of soil inorganic N availability resulted in a negative effect of N addition on NRE. Fire also led to a negative effect on plant NRE at community level, due to its negative effect on ANPP and positive effect on soil inorganic N. The negative effects of N addition and fire on community NRE suggests that part of soil inorganic N could not be taken up into plant biomass. Thus, more soil inorganic N would be lost by leaching and volatilization after N addition or burning. There is evidence that annually burning induce N loss through volatilization during fire [44], erosion, leaching, and denitrification [45]. It is notable that, the negative effects of N addition and fire on plant community NRE were independent, rather than additive. Together, our results indicate that the temperate steppe would be less efficient in absorbing the increased soil inorganic N into plant biomass after N addition and burning.

The dominant species examined in this study greatly varied in their N use traits. In the control plots, the NRE of *L. chinensis* was almost five times higher than that of *A. cristatum* and *C. squarrosa*. The higher NRE of *L. chinensis* would help explain its dominant status in this N-limited grassland ecosystem. More soil N would be transferred to plant biomass if this ecosystem was mono-dominated by *L. chinensis*, as indicated by its higher NRE status. Consequently, any changes of community composition following N addition and fire would influence plant N use at community level. Moreover, the responses of plant N economy to N addition and fire were species-specific. While N addition significantly enhanced the N uptake of *A. cristatum* and *C. korshinskyi*, it showed no impacts on that of other species. Similarly, fire reduced NRE of *A. cristatum* and *C. korshinskyi*, but had no effects on other species. The idiosyncratic responses of different species to N addition and fire suggest that *A. cristatum* and *C. korshinskyi* were more sensitive to changes of soil N availability resulting from N addition and annual burning. Our results have great implications for the plant-mediated changes of biogeochemical cycling in response to global changes and ecosystem management strategies.

We hypothesized that the responses of plant N use traits to N addition and fire would be different at species and community levels. When all the dominant species were analyzed together, we found that the responses of plant N concentrations and NRE to N addition and fire did not different ranging from species to community level. Nitrogen addition did not alter plant N concentrations at both species and community levels, whereas it did reduce plant NRE at both levels. Fire decreased plant N concentrations and NRE at community level, while its effects on species-level plant N concentrations and NRE varied among different species. However, when those species were analyzed individually, we did found divergent responses of plant N use traits at species and community levels, which would be mainly resulted from the species-specific responses of plants to N addition and fire. In a recent study, Iversen et al. [11] reported that plant N use traits differed at different biological organizations ranging from individual plant tissue to the entire community in peatland ecosystems. Our study presented strong evidence for the hierarchical responses of plant N use to N addition and fire at different biological scales, especially for species and community levels in a grassland ecosystem.

### Conclusions

Our results showed that both N addition and fire directly affected soil N availability and plant N use traits, with modest interaction between them. N addition increased ANPP, inorganic N, and N uptake, decreased NRE, and did not affect plant N concentrations. Prescribed fire, as an important management strategy in grasslands, enhanced plant N concentrations and decreased N uptake efficiency and NRE. The responses of plant N use to both N addition and fire would be different at species and community levels, mainly due to the species-specific responses. The divergence of plant N use traits among different plant species indicates that any factors leading to changes of community composition would indirectly alter plant N use at community level.
